# Clinical and histological evolution after *de novo* donor-specific anti-human leukocyte antigen antibodies: a single centre retrospective study

**DOI:** 10.1186/s12882-018-0886-5

**Published:** 2018-04-12

**Authors:** Yassine Bouatou, Olivia Seyde, Solange Moll, Pierre-Yves Martin, Jean Villard, Sylvie Ferrari-Lacraz, Karine Hadaya

**Affiliations:** 1 Division of Nephrology, Geneva University Hospitals, Rue Gabrielle Perret-Gentil 4, 1205 Geneva, Switzerland; 2 Institute of Clinical Pathology, Geneva University Hospitals, Geneva, Switzerland; 3 Immunology and Transplant Unit, Geneva University Hospitals, Geneva, Switzerland; 4 Division of Transplantation, Geneva University Hospitals, Geneva, Switzerland

**Keywords:** *de novo* DSA, Renal pathology, HLA-antibody post-transplantation, Outcome

## Abstract

**Background:**

Donor-specific anti-human leukocyte antigen (HLA) antibodies (DSA) can be preformed or *de novo* (*dn*). Strategies to manage preformed DSA are well described, but data on the management and outcomes of *dn*DSA are lacking.

**Methods:**

We performed a retrospective analysis of data from a single centre of the management and outcomes of 22 patients in whom a *dn*DSA was identified with contemporary and follow up biopsies.

**Results:**

Evolution from baseline to follow up revealed a statistically significant loss of kidney function (estimated glomerular filtration rate: 45.9 ± 16.7 versus 37.4 ± 13.8 ml/min/1.73 m^2^; *p* = 0.005) and increase in the proportion of patients with transplant glomerulopathy (percentage with cg lesion ≥1: 27.2% vs. 45.4%; *p* = 0.04). Nine patients were not treated at the time of *dn*DSA identification, and 13 patients received various drug combinations (e.g., corticosteroids, plasmapheresis, thymoglobulins and/or rituximab). No significant pathological changes were observed for the various treatment combinations.

**Conclusion:**

Our retrospective analysis of a small sample suggests that *dn*DSA should be considered a risk factor for the loss of kidney function independent of the baseline biopsy, and multidisciplinary evaluations of the transplant patient are a necessary requirement. Further confirmation in a multicentre prospective trial is required.

## Background

Kidney transplantation (KT) remains associated with suboptimal 5- and 10-year graft survival (77% and 56%, respectively [1]), despite an excellent 1-year allograft survival (91%). Graft failure is primarily associated with antibody mediated rejection (ABMR) [2–4].

Donor-specific anti-human leukocyte antigen (HLA) antibodies (DSA) drive ABMR. Development of the solid phase assay (SPA) to detect DSA was significant in the diagnosis of DSA [5]. SPA can detect DSA at the time of transplantation even with a negative flow cytometry crossmatch. DSA is divided into two categories: preformed DSA and *de novo* DSA (*dn*DSA). Preformed DSA may be associated with hyperacute rejection or ABMR in the weeks following transplantation. The presence of preformed DSA is associated with graft failure [6–8].

DSA development after KT is known as *dn*DSA. A previous review [9] noted that *dn*DSA primarily occurred during immunosuppression [10] in the context of poor adherence [4, 11, 12]. Early calcineurin inhibitor minimization may also be associated with *dn*DSA development [9]. Wiebe et al. [12] reported a trend towards an association with a history of acute rejection as an independent risk factor for *dn*DSA. *Dn*DSA is independently associated with poor long-term allograft outcomes [13]. SPA technique allows to further detect complement-binding DSA, and these DSAs are associated with reduced graft survival compared to non-complement-binding DSA [14, 15].

Knowledge of the presence of DSA at the time of transplantation allows implementation of specific therapeutic strategies (e.g., desensitization) to reduce the levels of preformed DSA [16]. Several studies demonstrated acceptable long-term patient survival compared to wait-listed patients on dialysis [16, 17]. However, the therapeutic management of *dn*DSA remains controversial. Plasma exchange, intravenous immunoglobulin (IVIG), anti-thymoglobulin antibodies (ATG), rituximab, bortezomib and treatment abstention are used in different combinations for the treatment of ABMR [18]. Data on the long-term efficacy of these regimens and treatment strategies guided by the presence of *dn*DSA (independent histology) are lacking.

The present study retrospectively analysed the diagnosis and clinical and histological evolution in patients who developed *dn*DSA.

## Methods

### Patient selection and follow up

We retrospectively analysed the stored sera available from all consecutive patients who underwent kidney transplantation in Geneva University Hospitals from 1986 to 2012. Patients with combined transplantation (kidney – liver, kidney – heart or kidney – pancreas) were excluded. The cumulative incidence of *dn*DSA is 15% in our centre. We identified 22 patients with known *dn*DSA from the 315 patients transplanted from 1986 to 2012 for whom we had available sera. *Dn*DSA was defined as a DSA that developed within six months after kidney transplantation with a mean fluorescence intensity (MFI) > 500. All 22 patients were transplanted with a negative CDC crossmatch (B and T) and/or FACS crossmatch. Clinical data were recorded during regular follow up at our institution. Graft function was assessed using the estimated glomerular filtration rate (eGFR). We used the CKD-EPI formula for each patient. The eGFR was calculated at the time of biopsy. A patient’s return to haemodialysis defined graft failure. We analysed graft biopsies at the time of *dn*DSA detection (± 3 months) in these patients and therapy administration according to the histological data. We identified a positive DSA in 2012 and retrospectively evaluated the sera from these patients to identify the first positive serum and matched the biopsy at the time of first identification of the *dn*DSA. The biopsies matched with the *dn*DSA were a protocol or indication biopsy because of the retrospective nature of this study.

The ethical committee approved the study (N° 6-208) which is in accordance with the regulations of the Geneva University Hospitals.

### Immunosuppression dnDSA/histological-based treatment

Induction therapy evolved over time and consisted of no induction or basiliximab on post-operative days (PODs) 0 and 4. Antithymocyte globulin (ATG) or daclizumab reflected the use of a steroid-free protocol in selected patients. The immunosuppressive regimen consisted of tacrolimus or cyclosporine A, corticosteroids, and mycophenolate mofetil, mycophenolic acid or azathioprine. We reported the identified treatment combination at the time of *dn*DSA and biopsy as reported in medical files. The reported combinations included high doses of steroids, ATG, anti-CD20 and/or plasma exchange. These treatments were adjusted according to histopathological findings and patient’s age and comorbidities (e.g., cancer, previous infections).

### Graft biopsies

Protocol biopsies are performed in our centre at one year post-transplantation, but the present analysis focused on kidney biopsies performed at approximately the time of development of *dn*DSA, regardless of kidney function. The clinician may not have known of the presence of *dn*DSA at the time of the biopsy. A control biopsy at the last follow up was also included to evaluate the histological changes following treatment of the *dn*DSA. The next available graft biopsy was used in patients who were not treated. Criteria from the Banff 2013 classification were used for the retrospective grading of biopsies [19].

### Determination of DSA using Luminex solid-phase assay

Patient sera were analysed for the presence of anti-HLA class I and class II antibodies using solid phase assays on Luminex and the Labscreen Mix assay for HLA class I and HLA class II following the recommendations of the manufacturer (One Lambda, Canoga Park, CA), as previously described [20]. Sera collected before and after transplantation of all positive individuals were subsequently tested for anti-HLA class I- and class II-specific antibodies using the Luminex single antigen beads (one Lambda). Briefly, colour-coded microspheres coated with major HLA class I and II antigens were incubated with 10 μl serum for 30 min at room temperature in the dark. Samples were washed three times and incubated with 100 μL of 1:100 phycoerythrin-conjugated goat anti-human IgG (One Lambda Inc.). Samples were washed twice, and the fluorescent signal intensity for each microsphere was measured using a LABScan 100 flow analyser (One Lambda Inc.). The cut-off for positive samples was the normalized background (NBG) ratio recommended by the manufacturer, which was performed using HLA Fusion software (One Lambda). An MFI > 500 was considered positive, as recommended by the manufacturer, and clinical relevance was considered at MFI > 1000. Donor-specific antigens were classified as immunogenic HLA or non-immunogenic HLA based on the presence of specific antibodies in the recipient directed towards the donor-specific antigens.

Post-transplantation screening consisted of Luminex solid-phase assays performed at 1 month, 3 months, 6 months, 9 months, 12 months and annually. Each increase in serum creatinine > 25% required a new Luminex solid-phase assay.

### Statistical analysis

Statistical analyses were performed using STATA v. 14.0 (College Station, TX). We used descriptive statistics to estimate the frequencies (%) and means (±SD) of study variables. Comparisons of two means were performed using *t* tests, and comparisons of the frequencies between groups were evaluated using Fisher’s exact test because of the small sample size. We did not perform further analyses because of the retrospective nature of the descriptive data.

## Results

### Patients’ characteristics

Table 1 summarises the patients’ characteristics. The characteristics of these patients were similar to the transplanted patients who do not develop *dn*DSA (data not shown). The mean age was 45 years (standard deviation (SD): ± 14), and males predominated (14/22; 63.6%). These kidney transplantations were primarily first ABO compatible, with greater than 3 HLA mismatches. A delayed graft function was observed in 8/22 patients (36.3%), and 8/22 patients (36.3%) did not receive induction therapy. Most patients who developed *dn*DSA received a triple immunosuppression regimen that was prescribed at baseline (21/22 patients; 95.4%). Eight of the 22 patients (36.3%) were treated for a previous episode of T-cell-mediated rejection (TCMR).

**Table 1:** Characteristics of patients and transplantations with late *de novo* DSA

	Patients with late *dn*DSA (*n* = 22)
*Recipients’ characteristics*	
Age in years; mean (SD)	45 (14)
Gender (% male)	63.6
First transplantation (%)	100
Living donor (% yes)	36.3
Baseline nephropathy (%)	
○ ADPKD	31.5
○ Glomerulopathy other than IgA nephropathy	18.3
○ IgA nephropathy	18.3
○ Diabetes mellitus and/or hypertension	13.6
○ Others	18.3
*Transplantation characteristics*	
HLA mismatches (% patients with > or = 3 mismatches)	100
ABO incompatible (n)	1
Delayed graft function (n, %)	8 (36.3)
Induction therapy (n, %)	
○ No induction	8 (36.3)
○ Basiliximab	8 (36.3)
○ ATG	5 (22.7)
○ Daclizumab	1 (4.5)
Initial therapy including a triple regimen^a^ (n, %)	21 (95.4)
Acute rejection or TCMR prior to development of *dn*DSA (n, %)	8 (36.3)

*ADPKD* Autosomal dominant polycystic kidney disease, *ATG* Antithymoglobulin, *SD* Standard deviation, *TCMR* T-cell-mediated rejection, *dnDSA* *de novo* DSA

^a^: defined by a combination of calcineurin inhibitor, anti-metabolite (azathioprine or mycophenolate mofetil or mycophenolic acid) and corticosteroids at one year

### Characteristics of the dnDSA

**Fig. 1:**
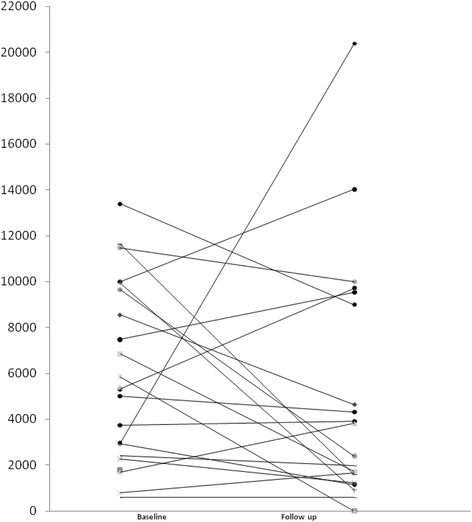
Individual evolution of *dn*DSA values in mean fluorescence intensity (MFI)

Table 2 describes the characteristics of the *dn*DSA. DSA was primarily directed against MHC class II antigens in 15/22 patients (68.1%) or in association with Class I *dn*DSA (6/22; 27%) with a mean MFI of 5896. No difference in the mean MFI for *dn*DSA was observed from baseline to follow up (Fig. 1). Notably, 18 patients (81.8%) received a triple immunosuppressant regimen one year post-kidney transplantation, but only 5 of the 22 patients (22.7%) received the triple therapy at the time of *dn*DSA detection. Less than 20% of the 22 patients with *dn*DSA exhibited concomitant acute kidney injury at the time of *dn*DSA, which was defined as an increase in creatinine ≥25% from baseline and/or appearance of proteinuria > 0.5 g/24 h at the time of *dn*DSA with a mean eGFR of 45.9 ml/min/1.73 m^2^ (SD: ± 16.7). Half of the patients exhibited albuminuria at the discovery of *dn*DSA.

**Table 2:** Characteristics of late *de novo* DSA (*dn*DSA)

	Patients with late *dn*DSA (*n* = 22)
Time from kidney transplantation to *dn*DSA in years; mean (SD)	10 (7.5)
HLA class (n, %)	
Class I	1 (4.5)
Class II	15 (68.1)
Class I + II	6 (27.0)
MFI mean (SD)	5896 (3879)
Triple immunosuppression 1 year post-Tx (n, %)	18 (81.8)
Triple immunosuppression at the time of *dn*DSA (%)	5 (22.7)
AKI at the time of *dn*DSA (n, %)	4 (18.2)
Mean eGFR using the CKD-EPI equation in ml/min (+/− SD)	45.9 (16.7)
Albuminuria at the time of *dn*DSA diagnosis (n, %)	12 (54.5)

*MFI* mean fluorescence intensity, *Tx* transplantation, *AKI* acute kidney injury, *eGFR* estimated glomerular filtration rate, *CKD-EPI* Chronic Kidney Disease Epidemiology Collaboration, *SD* standard deviation

### Histological parameters before and after therapy

**Fig. 2:**
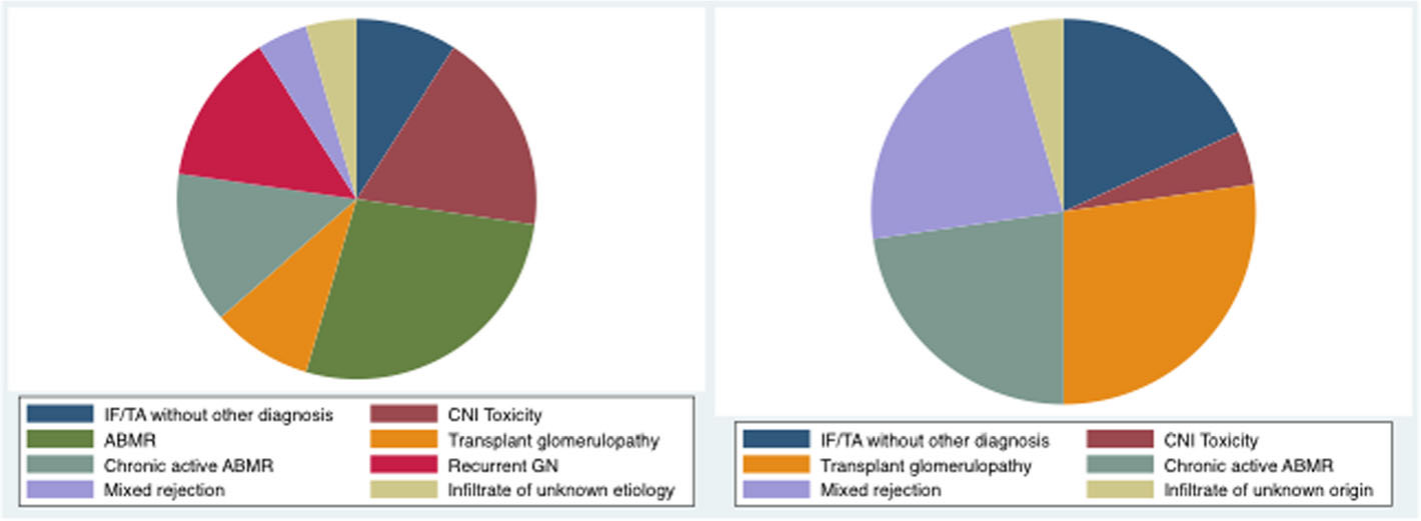
Final reported diagnosis at baseline and follow up biopsy. IF/TA: interstitial fibrosis with tubular atrophy; CNI: calcineurin inhibitor; ABMR: antibody mediated rejection; GN: glomerulonephritis

A median time of 55.5 days was observed between first *dn*DSA identification and the initial graft biopsy. The range was 102 days prior to the biopsy to 863 days after the biopsy. Figure 2 shows the diagnosis at the initial and the follow up biopsies. We observed that 12/22 (54.5%) patients had a diagnosis of acute ABMR, chronic ABMR or mixed rejection at the time of *dn*DSA.

Follow up biopsies occurred at a median of 812 days after the baseline biopsy (392; 1624). Most patients presented with chronic active ABMR and transplant glomerulopathy at the follow up biopsy (11/22; 50%).

We did not observe a significant change in microvascular inflammation (MVI) parameters (g, ptc separately, g + ptc) or C4d on two consecutive biopsies (Table 3). However, a significant increase in the proportion of patients with a chronic transplant glomerulopathy (cg) score ≥ 1 from 27.2% to 45.4% (*p* = 0.04) was observed between the two biopsies (Table 3).

**Table 3:** Overall clinical and histological changes over time (*n* = 22)

	Biopsy at the time of dnDSA	Follow up biopsy	Paired *t*-test
Mean eGFR using the CKD-EPI equation (ml/min, mean +/− SD)	45.9 (16.7)	37.4 (13.8)	0.005
g (n, % of g ≥ 1)	10 (45.4)	11 (50.0)	0.47
ptc (n, % of ptc ≥ 1)	10 (45.4)	6 (27.2)	0.26
g + ptc (n, % of g + ptc ≥ 2)	10 (45.4)	7 (31.8)	0.77
Overall change in g + ptc (n, %)			
○ Reduction in g + ptc score		9 (40.9)	
○ No change in g + ptc score		7 (31.8)	
○ Progression in g + ptc score		6 (27.3)	
t (n, % of *t* ≥ 1)	4 (18.1)	0	0.04
C4d (n, % of c4d ≥ 1)	9 (40.9)	5 (22.7)	0.16
cg (n, % of cg ≥ 1)	6 (27.2)	10 (45.4)	0.04

*eGFR* estimated glomerular filtration rate, *CKD-EPI* Chronic Kidney Disease Epidemiology Collaboration, *SD* standard deviation, *g* glomerulitis, *ptc* peritubular capillaritis, *t* tubulitis, *cg* chronic glomerulopathy

The eGFR dropped from a mean of 45.9 (SD: ± 16.7) at the first biopsy to 37.4 ml/min/1.73 m^2^ (SD: ± 13.8) at the follow up biopsy (Table 3; *p* = 0.005).

Thirteen of the 22 patients (59.1%) received a therapeutic regimen that included a steroid pulse (*N* = 8), plasma exchange (*N* = 6), IVIG (*N* = 5) and/or rituximab (*N* = 6). Evaluation of the overall effect of any treatment versus no treatment at the time of *dn*DSA on the evolution of MVI and cg score in the follow-up biopsy (Table 4) revealed no statistically significant difference. Treatment did not significantly influence the evolution of eGFR (Table 4).

**Table 4:** Overall effect of treatment on the evolution eGFR, microvascular inflammation (MVI) and chronic transplant glomerulopathy (cg); Fisher’s exact test *p* = 0.64, 0.86 and 1.0, respectively

	No treatment at the time of *dn*DSA (*n* = 9)	Any combination of treatment at the time of *dn*DSA (*n* = 13)
eGFR evolution		
○ eGFR increase	3	4
○ Stable eGFR	1	0
○ eGFR decrease	5	9
MVI evolution		
○ MVI reduction	3	6
○ No change in MVI	3	4
○ MVI progression	3	3
Cg evolution		
○ Cg reduction	1	0
○ No change in cg	5	11
○ Cg progression	3	2

*eGFR* estimated glomerular filtration rate, *CKD-EPI* Chronic Kidney Disease Epidemiology Collaboration, *MVI* microvascular inflammation defined as the g + ptc score

We analysed each specific drug and the evolution of MVI score (Table 5) or cg score (Table 6). Steroids were associated with a reduction in the MVI score (Trend only, *p* > 0.05), and IVIG therapy was associated with a progression of MVI score (*p* < 0.05). None of the other treatments were significantly associated with a modification of scores even considering a clinically relevant baseline score for MVI (Table 5) and cg (Table 6).

**Table 5:** Combination of treatment at the time of *dn*DSA (*n* = 13) and overall MVI outcomes (*Fisher’s exact test *p* < 0.05)

*N* = 13	Rituximab		Plasma exchanges		IVIG		Steroid bolus and treatment reinforcement	
	*yes*	*no*	*yes*	*no*	*yes*	*no*	*yes*	*no*
MVI reduction (*n* = 6)	3	3	3	3	0	6*	5	1
No change in MVI (=4)	1	3	1	3	3	1	2	2
MVI progression (*n* = 3)	2	1	2	1	2	1	1	2

*IVIG* intravenous immunoglobulins, *MVI* microvascular inflammation defined as g + ptc score

**Table 6:** Combination of treatment at the time of *dn*DSA (*n* = 13) and overall cg outcomes (*Fisher’s exact test *p* < 0.05)

*N* = 13	Rituximab		Plasma exchanges		IVIG		Steroid bolus and treatment reinforcement	
	*yes*	*no*	*yes*	*no*	*yes*	*no*	*yes*	*no*
No change in cg (*n* = 11)	5	6	4	7	4	7	6	5
Cg progression (*n* = 2)	1	1	2	0	1	1	2	0

*IVIG* intravenous immunoglobulins, *cg* chronic glomerulopathy

The mean graft survival was 13.8 years (± 7.8 years: range 6.0-21.6) from transplantation date to the last follow up, and no graft loss was observed.

## Discussion

Our retrospective analysis reports clinical and histological data from a single centre cohort of kidney transplant recipients who developed *dn*DSA. No improvement in histopathological parameters was observed with any therapeutic intervention. We observed a loss of kidney function and increase in cg score ≥ 1 at follow up. Graft survival was similar in patients who received treatment at the time of *dn*DSA and patients who did not receive therapy.

Wiebe et al. [12] analysed the clinical and histopathological correlations with *dn*DSA in 315 consecutive renal transplants without pre-transplant DSA. The mean follow-up was shorter (6.2 ± 2.9 years). Forty-seven of the 315 (15%) patients developed *dn*DSA 4.6 years post-transplant, which is earlier than our cases. They found that the two independent predictors of *dn*DSA were a mismatch for HLA-DRβ1 > 0 (OR 5.66, *p* < 0.006) and non-adherence (OR 8.75, *p* < 0.001). The authors demonstrated that the median 10-year graft survival for patients with *dn*DSA was lower than the no *dn*DSA group (57% vs. 96%, *p* < 0.0001). Their study found a non-significant trend with an odds ratio of 1.57 per rejection episode prior to an occurrence of *dn*DSA (*p* = 0.061). A high prevalence of TCMR episodes preceded the development of *dn*DSA (36.3%) in our study. Less than 20% of the 22 patients who developed *dn*DSA exhibited graft dysfunction at the time of diagnosis, which is consistent with Yamamoto et al., who reported a prevalence of 40% of patients with *dn*DSA and biopsy-proven subclinical ABMR [21]. Histological features at the time of *dn*DSA identification were not broadly consistent with ABMR in our study, and less than 50% of the biopsies exhibited an MVI score ≥ 2. C4d was negative in 59.1% of our baseline biopsies, which is consistent with previous reports [22, 23].

Further analyses of the incidence and impact of *dn*DSA in renal transplant recipients were performed. Forty-seven of the 189 consecutive non-sensitized, non-HLA-identical patients who received a kidney transplant between March 1999 and March 2006 (25%) developed *dn*DSA within 10 years [24]. The cumulative incidence 5 years post-transplantation was 20%, and more than half of these patients developed *dn*DSA in the first post-transplantation year. Patients of a younger age (< 35 years old), deceased donor, the presence of HLA antibodies (non-DSA) and DQ mismatch (as previously described [25]) were most likely to develop *dn*DSA in our study.

One strength of our analysis is the focus on the evolution of histological parameters in patients with *dn*DSA. Focusing on cases with negative crossmatch and DSA identified 6 months post-KT avoided the analysis of patients with preformed DSA. Our data evaluated the effect of each component of treatment on the evolution of the primary features of the Banff score associated with subtypes of ABMR [26]. Halloran et al. performed a recent principal component analysis of 164 indication biopsies [26] and found 3 main ABMR phenotypes: early ABMR with MVI lesions only (called pgABMR), late ABMR with cg lesions (cgABMR) and mixed feature (pgcgABMR).

The present study has several limitations. First, it used a retrospective analysis or different therapeutic regimens. However, we provide data on a broad heterogeneous kidney transplant recipient population that reflects routine clinical practice. Second, the small number of patients, without a control non-*dn*DSA group, prevents us from drawing any conclusions about the best protocol to treat *dn*DSA. We did not evaluate patient adherence, which is a known cause of *dn*DSA development and eventual graft failure [4]. Finally, an obvious pitfall was the non-standardisation of our treatment following *dn*DSA and biopsy results. The current evidence is insufficient to create an internationally approved protocol for the treatment of *dn*DSA. Our treatment protocol for ABMR evolved over time and reflects changes in practice based on the literature and a tailoring of therapy based on patient age, co-morbidities and cancer risk. Notably, the absence of IVIG as a component of the treatment given at the time of *dn*DSA was associated with MVI reduction. Current evidence is conflicting on the utility of IVIG in the treatment of antibody mediated injury. The benefit has been reported for active ABMR but its benefit on late/chronic ABMR is less straight forward [27].

Evidence supporting the use of rituximab for MVI or cg lesion is also scant [27, 28]. A recent randomized double-blind, placebo-controlled trial did not demonstrate a significant advantage of rituximab in biopsy-proven ABMR in creatinine level or proteinuria at 12 months [28]. The decision to treat *dn*DSA independently of histological features has not been addressed. Use of an intragraft transcript set measurement, as proposed in the 2013 Banff update [19], may further help the reclassification of these biopsies, but this methodology is not widely implemented yet [29].

## Conclusions

We describe retrospective results from a single centre of the clinical and histological features at the time of *dn*DSA collection with a follow up biopsy after therapeutic management. We observed no difference in histological evolution with or without treatment. Evolution was marked by a decrease in kidney function and increase in proportion of patients with transplant glomerulopathy at the follow up biopsy. No statistically significant changes in the MVI or cg parameters were observed in the follow up biopsy of patients who received different treatments. Further intervention studies are needed to prospectively evaluate the treatment stratification and integration of the *dn*DSA status, biopsy histological and molecular features and patient’s functional status and comorbidities. Novel drugs are necessary to control *dn*DSA and its long-term effect on graft function.

## Abbreviations

ABMR: Antibody mediated rejection
ATG: Antithymocyte globulin
CDC: Complement-dependent cytotoxicity
*dn*DSA: *de novo* donor specific antibody
eGFR: estimated glomerular filtration rate
HLA: Human leukocyte antigen
IVIG: Intravenous immunoglobulins
MVI: microvascular inflammation
POD: post-operative day
SPA: solid phase assay
TCMR: T-cell mediated rejection
